# Dysfunction of the Default Mode Network in Drug-Naïve Parkinson’s Disease with Mild Cognitive Impairments: A Resting-State fMRI Study

**DOI:** 10.3389/fnagi.2016.00247

**Published:** 2016-10-26

**Authors:** Yanbing Hou, Jing Yang, Chunyan Luo, Wei Song, Ruwei Ou, Wanglin Liu, Qiyong Gong, Huifang Shang

**Affiliations:** ^1^Department of Neurology, West China Hospital, Sichuan UniversityChengdu, China; ^2^Huaxi MR Research Center, Department of Radiology, West China Hospital, Sichuan UniversityChengdu, China

**Keywords:** Parkinson’s disease, mild cognitive impairment, default mode network, fMRI, functional connectivity

## Abstract

**Objective:** Cognitive impairments are common in Parkinson’s disease (PD) and can even occur in the early stages. The default mode network (DMN) is highly relevant for cognitive processes; however, it remains largely unknown if changes in the DMN connectivity are related to the cognitive decline in drug-naïve early stage PD patients with a mild cognitive impairment (MCI). This study used resting-state functional MRI (fMRI) to explore the brain connectivity of the DMN in early stage drug-naïve PD patients with MCI.

**Method:** We recruited 32 early stage drug-naïve PD patients and 22 matched healthy controls (HC). Among the PD patients, 14 were classified as having MCI (PD-MCI) and 18 were classified as having unimpaired cognition (PD-CU). The functional integration of the DMN was evaluated by a seed-based correlation approach.

**Results:** The brain connectivity analysis revealed reduced functional connectivity (FC) in both PD subgroups compared with HC. The PD-MCI group showed a significant reduction in FC between the DMN and a set of regions, including the precentral gyrus, middle temporal gyrus, insula, anterior inferior parietal lobule and middle frontal gyrus. Compared to the PD-CU group, the PD-MCI group demonstrated a significantly decreased FC in the middle frontal and middle temporal gyri. Additionally, compared to HC, the PD-MCI group had a significantly decreased FC within the DMN, mainly in the FC between the hippocampal formation and inferior frontal gyrus, between the posterior cingulate cortex and posterior inferior parietal lobule, and between the anterior temporal lobe and inferior frontal gyrus. Compared to the PD-CU group, the only significantly decreased FC within the DMN in the PD-MCI group was between the anterior temporal lobe and inferior frontal gyrus. In all PD patients, the decreased FC between anterior temporal lobe and middle temporal gyrus was positively correlated with attention/working performance, and the reduced FC between the hippocampal formation and inferior frontal gyrus was also positively correlated with memory function.

**Conclusion:** Our findings suggest that an altered DMN connectivity characterizes PD-MCI patients. These findings may be helpful for facilitating the further understanding of the potential mechanisms underlying MCI in PD. However, our results are preliminary, and further investigation is needed.

## Introduction

Parkinson’s disease (PD), as a common neurodegenerative disease, is characterized by cardinal motor symptoms, including tremors, rigidity, bradykinesia and postural instability, and numerous non-motor symptoms (NMS) (Jankovic, 2008; Luo et al., 2014). Cognition impairments are prevalent NMS in PD (Williams-Gray et al., 2007) and could gradually lead to PD-associated dementia (Emre et al., 2007). Some non-demented patients exhibit significant deficits in one or more cognitive domains and are referred to as having a mild cognitive impairment (MCI), which is common in PD, even in the early stage of the disease (Aarsland et al., 2009; Poletti et al., 2011), with estimates of prevalence as high as 55% in newly diagnosed PD patients (Janvin et al., 2003). In PD patients with MCI, cognitive dysfunction does not interfere considerably with daily life, but they are at a high risk of subsequently developing dementia (Aarsland et al., 2009; Litvan et al., 2012). A better understanding of MCI in PD would facilitate the early administration of pharmacological and non-pharmacological interventions.

Convergent evidence from functional brain imaging has suggested an association between changes in DMN connectivity and neuropsychological performance (van Eimeren et al., 2009; Wu et al., 2011; Tessitore et al., 2012; Wolf et al., 2012; Amboni et al., 2015; Baggio et al., 2015). The existence of a DMN in the human brain was first confirmed via positron emission tomography (PET) and then primarily via functional MRI (fMRI). A number of studies attempted to understand the components and function of the DMN, and its interaction with other resting state networks (Wu et al., 2011). Then, the core brain regions constituting the DMN were consistently identified, such as the medial prefrontal cortex (mPFC), posterior cingulate cortex (PCC), inferior parietal cortex and hippocampal formation (HF), which were characterized by increased basal activity during rest and decreased (“deactivated”) basal activity during cognitive tasks. The most consistent observation is that the DMN is highly relevant for cognitive processes (Raichle et al., 2001). This result was further supported by recent evidence in different neurodegenerative disorders, such as Alzheimer’s disease (AD), frontotemporal dementia and amyotrophic lateral sclerosis, where DMN alterations have been associated with patients’ clinical status and cognitive impairment (Zhou et al., 2010; Agosta et al., 2012; Tedeschi et al., 2012). Although the pathophysiology underlying cognitive deficits in PD remains not entirely clear, efforts should be made to explore the role of the DMN in cognitive dysfunction in PD (Buckner et al., 2008). Previous studies have found that major nodes of the DMN, such as the mPFC and PCC, demonstrated significant dysfunction in PD patients in the resting state (Gorges et al., 2015) and while performing executive tasks (van Eimeren et al., 2009). However, little is known about the changes in DMN connectivity related to cognitive decline in drug-naïve early stage PD patients with MCI. Examining the DMN as an integrative network can provide new insights into the large-scale neural communication in the brain of PD patients with MCI.

Resting-state fMRI is a cognitively unbiased approach to explore functional brain connectivity (Raichle et al., 2001). This method demands minimal patient compliance and is relatively easy to implement in clinical studies. This technique has been successfully used to detect abnormal functional integration in PD. However, previous neuroimaging experiments were conducted on patients who had been chronically exposed to anti-Parkinson medications (Amboni et al., 2015), and even performed in the ON state (Tessitore et al., 2012). Chronic exposure to anti-Parkinson medications may result in the reorganization of functional integration, which might not reflect the primary pathophysiological changes induced by PD (Buhmann et al., 2003). In addition, dopaminergic therapy has been reported to have a dose-dependent effect on the DMN integrity in PD patients, such as generating an enhanced functional connectivity (FC) of the DMN in the PCC (Krajcovicova et al., 2012). Therefore, compared with studies on patients chronically exposed to anti-Parkinson medications, studies on drug-naïve PD patients may be critical to elucidate the role of the DMN in PD with MCI.

Our goal in this study was to use resting-state fMRI to characterize connectivity changes in the DMN in early stage drug-naïve PD patients with and without MCI, compared with a cohort of normal controls, using *a priori* seed-based analysis. We also aimed to assess the relationship between these changes in the patterns of network connectivity and the performance of cognitive functions frequently affected in PD, that is, attention/working memory, executive, memory, language and visuospatial (VS).

## Materials and Methods

### Participants

The local research ethics committee approved this study, and written informed consent was obtained from all of the participants. Patients who were recruited consecutively in our cohort were first diagnosed at the Movement Disorders Outpatient Clinic of West China Hospital of Sichuan University. These patients did not take anti-Parkinson medicines (i.e., drug-naïve) since they were not diagnosed correctly before visiting us. The recruited patients fulfilled the PD Society Brain Bank diagnostic criteria (Hughes et al., 1992). From this cohort, patients were excluded if they had (1) head motion being visible to the naked eyes; (2) cerebrovascular disorders, including previous stroke, history of head injury, history of seizure, hydrocephalus, intracranial mass, previous neurological surgery and other neurologic diseases; (3) anti-Parkinson, antidepressant or neuroleptic treatment before enrollment; and (4) history of dementia. After enrollment, the patients were followed-up for at least 1 year to confirm the diagnosis of PD because patients with PD at enrollment were in the early stage. Additionally, 22 normal subjects [healthy controls (HC) group] with no history of neurologic or psychiatric diseases were recruited and matched for age, gender and education with the PD subjects.

Movement disorder specialists collected data pertaining to handedness, age, sex, disease duration, and education years, and evaluated clinical symptoms using rating scales prior to MRI examination. The severity of PD was assessed using the Hoehn & Yahr staging scale (H&Y) and the Unified PD Rating Scale (UPDRS). The Hamilton Depression Rating Scale (HDRS) and the Hamilton Anxiety Rating Scale (HARS) were used to quantify depression and anxiety, respectively. The Montreal Cognitive Assessment (MoCA) was used to assess global cognitive function. Moreover, all participants underwent a complete neuropsychological battery. Attention/working memory [adaptive digit ordering test (DOT-A) and Golden Stroop test], executive [verbal fluency test (VFT) and clock drawing test (CDT)], memory [Hopkins verbal learning test-revised (HVLT-R) and brief visuospatial memory test revised (BVMT-R)], language [Wechsler intelligence scale for adults-Chinese revised (WAIS-RC) and Boston naming test (BNT)] and VS [Benton line orientation (BLO) and clock copying test (CCT)] functions were tested in all subjects. The cognitive performance scores were converted to *z*-scores and adjusted for age, sex, and education. Patients were classified as PD-MCI according to the new criteria of the Movement Disorder Society Task Force for mild cognitive impairment in PD. The criterion was met when patients performed approximately 1–2 standard deviation below the normative mean score on at least two neuropsychological tests, represented by either two impaired tests in one cognitive domain or one impaired test in two different cognitive domains (Litvan et al., 2012). In the current study, the PD-CU patients were defined as having a cognitive performance with normal scores or with abnormal scores with less than 1 standard deviation below the normative mean score. A total of 14 patients diagnosed as having mild cognitive impairment (PD-MCI group) with multiple domains and 18 patients diagnosed as cognition unimpaired (PD-CU group) were enrolled. PD-MCI patients were matched with PD-CU patients for PD disease severity. We arranged the enrolled patients to undergo the clinical evaluation and MRI scanning as soon as possible (usually on the second day after making a diagnosis of PD). The clinical assessment and MRI scanning were conducted on the same day prior to the initiation of any treatment. Then the patients started the anti-Parkinson treatment (usually on the same day after the clinical evaluation and MRI scanning).

### MRI Acquisition

An MRI was performed on a 3.0 Tesla MRI System (Siemens 3.0 T Trio Tim, Germany) by using an eight-channel phased-array head coil. High-resolution T1-weighted images were acquired via a volumetric three-dimensional spoiled gradient recall sequence. The acquisition parameters were as follows: repetition time [TR] = 1900 ms, echo time [TE] = 2.26 ms, flip angle [FA] = 90°, field of view [FOV] = 256 mm × 256 mm, slice thickness = 1 mm, no slice gap, voxel size = 1.0 mm × 1.0 mm × 1.0 mm, number of slices = 176. The MR images sensitized to changes in the BOLD signal levels (TR = 2000 ms, echo time = 30 ms, flip angle = 90°) were obtained via a gradient-echo echo-planar imaging sequence (EPI). The slice thickness was 5 mm (no slice gap) with a matrix size of 64 × 64 and a field of view of 240 mm × 240 mm, resulting in a voxel size of 3.75 mm × 3.75 mm × 5 mm. Each brain volume comprised 30 axial slices, and each functional run contained 240 image volumes. We provided ear plugs to each subject to reduce the noise interference during scanning and used foam to fix the participants’ head position and avoid head motion. The fMRI scanning was performed in darkness, and the participants were explicitly instructed to relax, close their eyes and not fall asleep (confirmed by subjects immediately after the experiment) during the resting-state MR acquisition.

### Preprocessing of fMRI Data

The functional image preprocessing and statistical analysis were conducted by using SPM^1^. The first 10 volumes of functional images were discarded for the signal equilibrium and participant adaptation to the scanning noise. The remaining EPI images were preprocessed via the following steps: slice timing, motion correction, spatial normalization to the standard Montreal Neurological Institute (MNI) EPI template in SPM8, and resample to 3 mm × 3 mm × 3 mm, followed by spatial smoothing with a 6 mm full-width at half-maximum (FWHM) Gaussian kernel. In our study, the scrubbing was not performed. However, the headers are modified for each of the input images, which can also reflect the relative orientations of the data. Then the details of the transformation are displayed in the results window as plots of translation and rotation, which can be modeled as confounds within the general linear model. In addition, according to the record of head motions within each fMRI run, all participants had less than 0.5 mm maximum displacement in the x, y, or z plane, and less than 0.5° of angular rotation about each axis.

### Functional Connectivity Analysis

Functional connectivity was examined by a seed-based correlation approach. Thus, we selected the following areas as seeds in the DMN: anterior, dorsal and ventral medial prefrontal cortex (mPFC), superior frontal gyrus (SFG), inferior frontal gyrus (IFG), posterior inferior parietal lobule (pIPL), precuneus, PCC, temporal parietal junction, anterior temporal lobe (ATL), superior temporal sulcus and HF. We defined the 18 nodes using the MNI coordinates (Spreng et al., 2013) (see **Supplementary Table S1**) and 10 mm radius circular masks. Using REST^2^, after bandpass filtering (0.01–0.08 Hz) and linear trend removal, the reference time series for each seed region was extracted by averaging the fMRI time series of all voxels within each region of interest. Correlation functional analyses were performed by computing the temporal correlation between each seed reference and the rest of the brain in a voxel-wise manner. Eight nuisance covariates were regressed to remove the possible variances from the time course of each voxel, including the white-matter signal, the cerebrospinal fluid (CSF) signal and six head motion parameters. The global signal regression was not included, because it is a controversial issue (Murphy et al., 2009). However, the results with global signal regression were included in the **Supplementary Table S2** and **Supplementary Figure S1**, and they were largely overlapped with the results without global signal regression. The correlation coefficients in each voxel were then transformed to *z*-value images by using the Fisher r-to-z transformation to improve normality. Hence, an entire brain *z*-value map was created for each subject.

In addition, to characterize the abnormal FC within the DMN, we chose these 18 nodes as being representative of the DMN and performed an additional region-wise manner. The mean-time series were extracted by the same way as above and were correlated between these 18 nodes of each subject. A Fisher’s r-to-z transformation was applied to normalize the correlation coefficient. Finally, the *z*-value was extracted for each participant, and we then compared *z*-values between the groups.

### Statistical Analysis

Differences between groups in terms of demographic and clinical variables were performed by the Pearson χ^2^ test, one-way analysis of variance (ANOVA), or Student’s *t*-test, as appropriate. A voxel-based comparison of *z*-value maps among the PD patients with MCI, PD patients with CU, and HC was performed by using a design model of one-way ANOVA with age, sex and education as covariates, followed by *post hoc* two-sample *t*-tests. The significance threshold was set at *p* < 0.001. Family-wise error (FWE) correction for multiple comparisons was also conducted at the cluster level. In addition, a comparison of the correlation values (*z*-values) among groups was performed by one-way ANOVA, followed by *post hoc* two-sample *t*-tests (*p* < 0.05, two-tailed). To explore the possible relationship between changes in the patterns of network FC and performance in cognitive functions, we conducted Pearson correlations between FC values of significant inter-group differences and cognitive scores, and the significance was set at *p* < 0.05 (two-tailed). FC values of significant inter-group differences were required from two ways of FC analyses. One is the voxel-wise analysis, and the statistical analysis model was one-way ANOVA among the three groups, followed by *post hoc* two-sample *t*-tests. The significance threshold was set at *p* < 0.001, and family-wise error (FWE) correction for multiple comparisons was performed at the cluster level. The other one is the region-wise analysis. We only chose the FC values showing significant inter-group differences within the DMN in the Pearson correlation analysis. Then, the Pearson correlation analyses were performed between these FC values of significant inter-group differences and cognitive scores in all PD patients.

### Voxel-Based Morphometry Analysis

In our study, diffeomorphic anatomic registration through an exponentiated lie algebra algorithm (DARTEL) (Ashburner, 2007) was used to improve registration of the MR images. Before segmentation, scanner artifacts and gross anatomic abnormalities for each subject were checked, and the image origin was set to the anterior commissure. Images were then segmented into gray matter (GM), white matter and CSF using the unified segmentation model in SPM8. In the next step, a GM template was generated through an iteratively non-linear registration. The GM template was normalized to MNI space, and the resulting deformations were applied to the GM images of each participant. Finally, spatially normalized images were modulated to ensure that the overall amount of each tissue class was not altered by the spatial normalization procedure and smoothed with a 10 mm FWHM Gaussian kernel. Voxel-based comparisons of GM volume were performed between groups using two-sample t-tests with age, sex and education as covariates. The significance thresholds were at voxel-wise *p* < 0.001 and the cluster level of *p* < 0.05 corrected by FWE correction.

## Results

### Demographic and Clinical Characteristics

The demographic and clinical features of all subjects are listed in **Table 1**. Handedness, age and sex were not significantly different among the HC, PD-MCI and PD-CU groups. Education was not significantly different between total PD patients and controls, whereas, the PD-CU group had significantly more education years than the PD-MCI group. No significant differences in the disease duration, H&Y stage, UPDRS score and HDRS/HARS score between PD-CU and PD-MCI groups were found. By definition, the MoCA scores of PD-CU patients were significantly higher than those of PD-MCI patients (*p* < 0.000). The scores of all neuropsychological tests of all subjects are provided (**Supplementary Table S3**).

**Table 1:** Demographic and clinical characteristics of all subjects.

Parameter	PD, all	Controls	PD-CU	PD-MCI	*P-value*^1^	*P-value*^2^	*P-value*^3^
Number, *n*	32	22	18	14	–	–	–
Handedness of writing (R: L)	32: 0	22: 0	18: 0	14:0	1	1	–
Age, *y*	54.16 ± 8.34	52.64 ± 6.65	53.61 ± 8.68	54.86 ± 8.13	0.479	0.705	0.682
Gender, M/F	14/18	9/13	9/9	5/9	0.839	0.717	0.435
Type of motor symptom (TD/PIDG/mixed)	18/11/3	–	10/7/1	8/4/2	–	–	–
Duration of disease, *y*	1.41 ± 1.14	–	1.53 ± 1.22	1.24 ± 1.05	–	–	0.485
H & Y stage (I: II: III)	–	–	8: 10: 0	3: 9: 2	–	–	0.071
UPDRS score	–	–	–	–	–	–	–
Part I- nM-EDL	–	–	1.06 ± 1.39	0.86 ± 1.17	–	–	0.671
Part II- M-EDL	–	–	5.56 ± 2.30	5.79 ± 3.64	–	–	0.829
Part III- motor examination	–	–	15.35 ± 5.58	17.78 ± 8.46	–	–	0.354
Part IV- motor complications	–	–	0	0	–	–	–
HDRS score	–	–	5.67 ± 6.62	6.43 ± 3.25	–	–	0.696
HARS score	–	–	4.39 ± 4.73	4.79 ± 3.68	–	–	0.798
MoCA score	–	–	27.61 ± 2.20	22.36 ± 2.56	–	–	<0.001^∗^
EDU, *y*	9.63 ± 3.48	9.55 ± 3.57	11.50 ± 3.22	7.21 ± 2.05	0.936	0.002^∗^	–


*^∗^Indicates significant difference; ^1^Comparison between all PD patients (PD-MCI and PD-CU) and control subjects. ^2^Comparison among PD-MCI, PD-CU patients, and control subjects. ^3^Comparison between PD-CU and PD-MCI patients. PD, Parkinson’s disease; PD-CU, cognitively unimpaired PD patients; PD-MCI, cognitively impaired PD patients; TD = Tremor dominant; PIDG = postural instability gait difficulty; H & Y, Hoehn & Yahr staging; UPDRS, Unified Parkinson’s Disease Rating Scale; nM-EDL, Non-Motor Aspects of Experiences of Daily Living; M-EDL, Motor Aspects of Experiences of Daily Living; HDRS, Hamilton Depression Rating Scale; HARS, Hamilton Anxiety Rating Scale; MoCA, Montreal Cognitive Assessment; EDU, Education.*

### FC Analysis in the DMN

**FIGURE 1:**
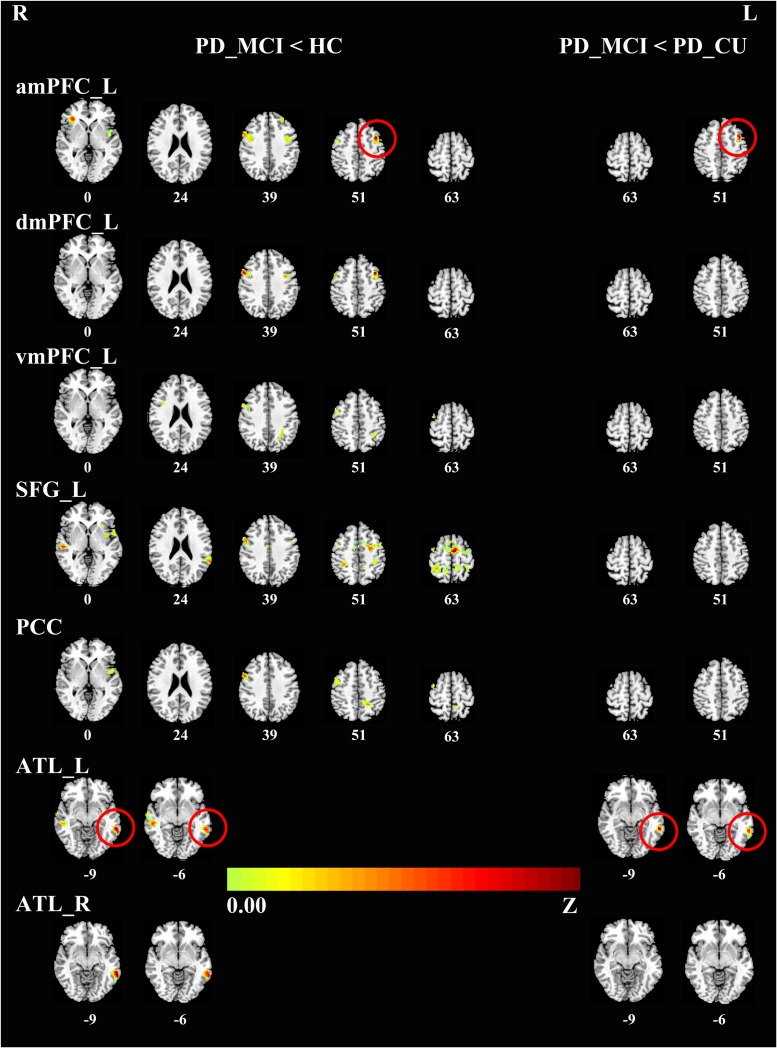
**Differences in the connectivity patterns are shown between PD-MCI and HC **(Left side)**, PD-MCI and PD-CU **(Right side)** (*p* < 0.001, FWE-corrected) in hot color.** The regions with red circles mean the same areas between the left side and the right side. PD, Parkinson’s disease; PD-MCI, mild cognitive impaired PD patients; PD-CU, cognitive unimpaired PD patients; HC, healthy control; amPFC, anterior medial prefrontal cortex; dmPFC, dorsal medial prefrontal cortex; vmPFC, ventral medial prefrontal cortex. SFG, superior frontal gyrus; PCC, posterior cingulate cortex; ATL, anterior temporal lobe; L, left; R, right.

To characterize the abnormal FC in the DMN, we chose 18 seed regions as representatives of the DMN. Relative to HC, the PD-MCI group showed significantly reduced connectivity of the DMN with a set of regions in the brain cortex. Specifically, the key hubs, mPFC, SFG and PCC, showed a decreased connectivity pattern with the right precentral gyrus and bilateral insula. Also, the mPFC and SFG showed a reduced coupling with the anterior inferior parietal lobule (aIPL); the mPFC and PCC showed a reduced coupling with the bilateral middle frontal gyri (MFG). FC analysis of the bilateral ATLs also showed a decreased connectivity pattern with the bilateral middle temporal gyri (MTG). The PD-MCI group presented significantly reduced connectivity of the DMN with the MFG and MTG compared to the PD-CU group. There was no significant difference between the PD-CU group and HC (see **Table 2**, **Figure 1**).

**Table 2:** Differences in functional connectivity among PD subgroups and normal subjects.

Seed area	*P*-value^1^	Size	Connected location	T	MNI coordinate (*x, y, z*)		
**HC group > PD-MCI group**					
Left anterior medial prefrontal					
Cluster 1	0.000	222	Left middle frontal	5.98	-36	12	51
Cluster 2	0.000	210	Right precentral	4.77	54	9	39
Cluster 3	0.004	141	Right insula	5.23	33	30	0
Cluster 4	0.011	111	Left insula	3.86	-45	-3	0
Cluster 5	0.049	77	Left middle frontal	4.25	-60	9	9
Left dorsal medial prefrontal					
Cluster 1	0.004	133	Right precentral	4.71	54	9	39
Cluster 2	0.007	120	Left middle frontal	4.88	-39	9	54
Left ventral medial prefrontal					
Cluster 1	0.003	141	Right precentral	4.00	54	9	39
Cluster 2	0.014	105	Left inferior parietal	3.90	-33	-48	51
Left superior frontal					
Cluster 1	0.000	797	Right precentral	5.19	51	6	36
Cluster 2	0.000	350	Right inferior parietal	4.65	33	-42	54
Cluster 3	0.000	240	Left insula	4.97	-39	-6	-21
Cluster 4	0.000	196	Left precuneus	4.52	-15	-42	60
Cluster 5	0.007	114	Right superior temporal	4.88	57	-24	3
Cluster 6	0.012	100	Left superior temporal	4.63	-63	-48	24
Posterior cingulate cortex					
Cluster 1	0.000	236	Right precentral	5.58	54	9	36
			Right middle frontal	4.42	42	0	60
Cluster 2	0.006	121	Left precuneus	4.70	-9	-45	57
Cluster 3	0.006	119	Left insula	3.83	-45	0	0
Left anterior temporal					
Cluster 1	0.005	135	Right middle temporal	4.58	54	-30	-6
Cluster 2	0.016	104	Left middle temporal	5.17	-63	-42	-9
Right anterior temporal					
Cluster 1	0.013	109	Left middle temporal	5.73	-66	-48	-9
**PD-CU group > PD-MCI group**					
Left anterior medial prefrontal					
	0.043	80	Left middle frontal	4.93	-39	9	51
Right anterior temporal					
	0.032	87	Left middle temporal	5.62	-63	-42	-9


*^1^*P*-value was corrected for multiple comparisons by family-wise error (FWE) correction. MNI, Montreal Neurological Institute.*

**FIGURE 2:**
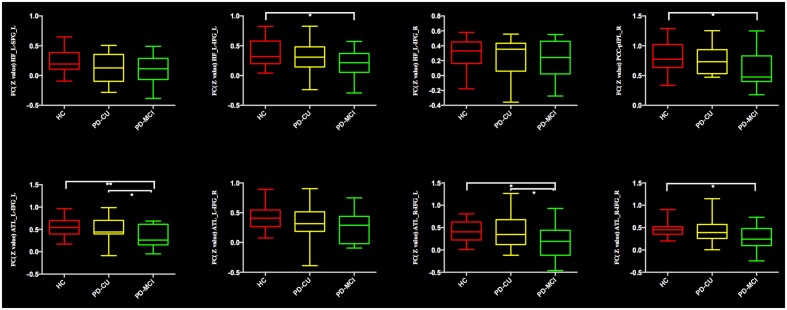
**Significantly reduced functional connectivity in the PD-MCI group is indicated by an asterisk (^∗^*p* < 0.05; ^∗∗^*p* < 0.01).** PD, Parkinson’s disease; PD-MCI, mild cognitive impaired PD patients; PD-CU, cognitive unimpaired PD patients; HC, healthy control; HF, hippocampal formation; SFG/IFG, superior/inferior frontal gyrus; ATL, anterior temporal lobe; PCC, posterior cingulate cortex; pIPL, posterior inferior parietal lobule; L, left; R, right.

Comparing the mean connectivity values, ordered reductions (HC > PD-CU > PD-MCI) were observed in intra-DMN connectivity, although there was no significant difference between the PD-CU group and HC. Compared with HC, the PD-MCI group showed significant intra-DMN reductions predominantly between HF and IFG, between ATL and IFG, and between PCC and pIPL. The PD-MCI group showed a significantly lower FC between ATL and IFG than the PD-CU group. The means and SDs of *z*-values are shown in **Figure 2** and **Supplementary Table S4**.

**FIGURE 3:**
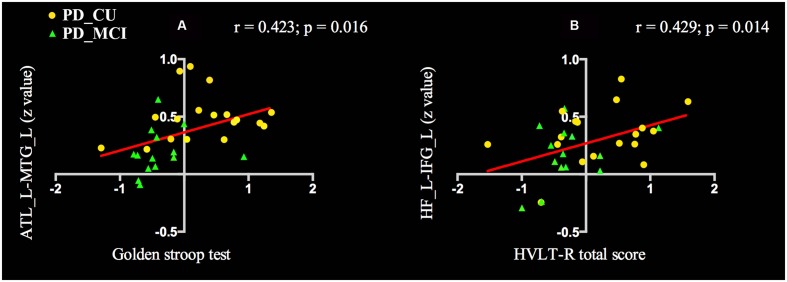
**Voxel-wise correlation analysis revealed that cognitive scores in PD patients were correlated with differential functional connectivity.** **(A)** The FC of ATL and MTG was positively correlated with Golden Stroop Test score. **(B)** The FC of HF and IFG was significantly correlated with the HVLT-R total score. PD, Parkinson’s disease; PD-MCI, mild cognitive impaired PD patients; PD-CU, cognitive unimpaired PD patients; ATL, anterior temporal lobe; MTG, middle temporal gyrus; HF, hippocampal formation; IFG, inferior frontal gyri; L, left; R. right; HVLT-R, Hopkins verbal learning test-revised.

To determine the relationship between the FC values and neuropsychological scores of PD patients, we performed the analysis in all PD patients. The FC of ATL and MTG was positively correlated with Golden Stroop Test score (a: *r* = 0.423, *p* = 0.016), and the functional coupling of HF and IFG was significantly correlated with the HVLT-R total score (b: *r* = 0.429, *p* = 0.014) (see **Figure 3**).

There were no significant differences among the three groups in the GM volume, indicating that the altered FC was not caused by anatomic changes.

## Discussion

In this study, we investigated the resting-state FC of the DMN in PD patients with and without MCI. Compared with HC, the PD-MCI group had a distributed reduction in connectivity between the DMN and a set of regions, including the precentral gyrus, insula, aIPL, MFG and MTG. Compared to the PD-CU group, the PD-MCI group demonstrated a significantly decreased FC in the MFG and MTG. Additionally, compared to HC, the PD-MCI group had a significantly decreased FC within the DMN, mainly in the FC between the HF and IFG, between ATL and IFG, and between PCC and pIPL. Compared to the PD-CU group, the only revealed significantly decreased FC within the DMN in the PD-MCI group was between the ATL and IFG. The decreased FC between ATL and MTG was positively correlated with attention/working performance. And the reduced FC between HF and IFG was also positively correlated with memory function. By studying a cohort of drug-naïve PD patients in the early stage, we ruled out potential confounding effects of chronic duration and medication.

Neuroimaging studies have demonstrated that a set of dynamically interrelated brain intrinsic connectivity networks is considered to play an important role in cognitive processing, including the DMN, dorsal attention network (DAN) and frontoparietal network (FPN) (Seeley et al., 2007; Spreng et al., 2013). The DAN activity was reduced whereas DMN activity was increased during the resting state, and the opposite was observed during cognitive tasks (Kelly et al., 2008). The FPN is interposed between the main DMN and DAN nodes in terms of anatomy and function, and can flexibly connect to one network or the other and mediate the transition between them (Vincent et al., 2008; Spreng et al., 2013). Activity in the DAN and FPN is high when attention is directed externally, associated with the reduction in activity within the DMN. These networks show anti-correlated activity, supporting that the DMN has widespread connections to the DAN and FPN, which has been suggested to be important for efficient cognitive function. In amnestic MCI, degenerated FC in DAN was revealed and the degenerations of the dorsal attention system were along with the progression of the disease and behavioral deficits in attention function (Zhang et al., 2015). In addition, neuroimaging studies have also consistently shown that working memory performance is associated with the FPN, whose task-related activity is increased in PD (Trujillo et al., 2015). Nevertheless, previous studies have mainly focused on changes affecting a single network; thus, little is known about how changes in inter-network connectivity are related to cognitive decline.

In the current resting state study, PD-MCI subjects displayed decreased FC between the DMN and regions of the precentral gyrus and MTG, which belong to DAN, as well as regions of the insula, aIPL and MFG which belong to the FPN (Spreng et al., 2013). Moreover, the connectivity between ATL and MTG was observed to positively correlate with attention/working performance in the PD subjects, indicating that reduced connectivity was associated with worse performance. In early AD, negative correlations between two intrinsically anti-correlated networks have been observed, suggesting that the disturbance of the anti-correlation might be associated with the attention deficits of AD patients (Wang et al., 2007). Moreover, for normal subjects, a higher magnitude of this anti-correlation was associated with improved behavioral attention responses (Kelly et al., 2008). Taken together, it could be speculated that this decreased FC between the DMN and a set of regions associated with DAN or FPN might be related to an impaired anti-correlation mechanism. However, it was rarely reported and is worth further research.

Aside from the decreased inter-network connectivity, we also observed reduced intra-DMN connectivity, inline with recent resting-state fMRI studies in medicated PD patients (Tessitore et al., 2012; Baggio et al., 2015; Gorges et al., 2015). In the current study, connectivity changes between the HF and IFG were positively correlated with memory performance. In the past decades, studies have focused on the hippocampus and related medial temporal lobe structures for their crucial roles in memory processes. The hippocampus has been reported to be atrophied and to be pathologically involved in both patients with MCI and patients in the early stage of AD (Braak and Braak, 1991; Desikan et al., 2009). Thus, HF was believed to have a close relationship with many cerebral cortexes and constitute the memory network to modulate and facilitate communication, which made it especially meaningful in MCI. Previous studies demonstrated a disrupted connectivity between the hippocampus and a set of regions, including the frontal lobe, temporal lobe and insula in MCI (Wang et al., 2011). Moreover, over half of the patients with PD dementia (PDD) also have significant AD-related pathology with abnormally deposited α-synuclein, amyloid-β and tau proteins in the PDD brain, including in the hippocampus which correlates to the severity of cognitive decline in patients with PDD (Kalaitzakis and Pearce, 2009). Our findings suggest that the change in connectivity between the hippocampus and frontal lobe might be part of the substrate of memory deficits in early stage drug-naïve PD with MCI. Interestingly, we also found decreased FC between ATL and IFG. The ATL, with a high degree of anatomical heterogeneity, makes a critical contribution to semantic cognition (Hurley et al., 2015; Jackson et al., 2016). In addition, semantic cognition depends on a distributed network including the IFG, mPFC, posterior middle temporal gyrus and lateral parietal regions besides ATL (Jefferies, 2013) and is also considered a major component during the resting state (Binder et al., 2009). Our observation may provide the underlying mechanism for the progressive semantic cognitive decline in the early stage PD-MCI group. As to the PCC, a critical node in the DMN, reduced connectivity with pIPL was identified in patients with PD-MCI in our study, which was consistent with results from a previous study in MCI patients (Wang et al., 2012). It is unclear how the PCC changes engaged in cognitive decline in PD-MCI, and further studies are needed to elucidate it.

One limitation of our study is the relatively small sample size of PD patients and HC. However, this limitation is partly due to the stringent quality control of clinical, neuropsychological, and imaging assessment, which in turn can be identified as a major strength of the study. Moreover, it was a possible explanation for the absence of any disruption in DMN associated with language and VS performance. Moreover, another limitation refers to the subjects’ education level where the PD-MCI group was less educated compared to the PD-CU group. The higher level of education is generally associated with a better cognitive reserve and is thought to provide the ability to recruit additional resources to compensate for brain damage (Stern, 2002). Although education was added as a covariate in the analyses, the influence of the difference of education levels cannot be entirely ruled out. In addition, the data are cross-sectional, and the mechanisms by which these network alterations change dynamically remain to be established in longitudinal studies.

## Conclusion

This study shows that mild cognitive decline in early stage drug-naïve PD is associated with a loss of inter-network connectivity and a decrease in the connectivity within the DMN. Moreover, our results provide support to the hypothesis that the DMN plays a role in the neural processing of distinct neuropsychological functions. This study represents a first step toward examining the functional integrity of the DMN in PD patients with MCI by using resting fMRI. However, our results are preliminary, and further longitudinal studies are needed to investigate whether early specific connectivity dysfunctions in the DMN could represent an early potential predictor for cognitive decline in PD. In addition, the neural substrate of cognitive decline in PD is not completely understood and is a matter of ongoing research.

## Author Contributions

HS and QG planned the study. YH, JY, CL, WS, RO, WL, and HS collected and analyzed clinical data, and made patient follow-ups. YH and JY conducted the imaging studies. YH wrote the article, and HS edited the paper.

## Conflict of Interest Statement

The authors declare that the research was conducted in the absence of any commercial or financial relationships that could be construed as a potential conflict of interest.

## Footnotes

1 http://www.fil.ion.ucl.ac.uk

2 http://www.restfmri.net/forum/REST_V1.7

## Supplementary Material

**FIGURE S1 | Differences in the connectivity patterns with global signal regression are shown between PD-MCI and HC (Left side), PD-MCI and PD-CU (Right side) (p < 0.001, FWE-corrected) in hot color.** The regions with red circles mean the same areas between the **Table 2** and the Supplementary Table S2. PD, Parkinson’s disease; PD-MCI, mild cognitive impaired PD patients; PD-CU, cognitive unimpaired PD patients; HC, healthy control; amPFC, anterior medial prefrontal cortex; dmPFC, dorsal medial prefrontal cortex; vmPFC, ventral medial prefrontal cortex. SFG, superior frontal gyrus; PCC, posterior cingulate cortex; IFG, inferior frontal gyrus; ATL, anterior temporal lobe; ST, superior temporal; L, left; R, right.

**TABLE S1 | Anatomical regions used as DMN nodes for seed-based connectivity analyses.**

**TABLE S2 | Differences in functional connectivity among PD subgroups and normal subjects with global signal regression.**

**TABLE S3 | Neuropsychological performance results for healthy controls and Parkinson’s disease patients.**

**TABLE S4 | Means and SDs of z values for all participants.**
