# Supplementary figures and images for: Dysfunction of the Default Mode Network in Drug-Naïve Parkinson’s Disease with Mild Cognitive Impairments: A Resting-State fMRI Study
Source: Front Aging Neurosci. 2016 Oct 26;8:247. doi: 10.3389/fnagi.2016.00247 (PMC5080293; doi:10.3389/fnagi.2016.00247)

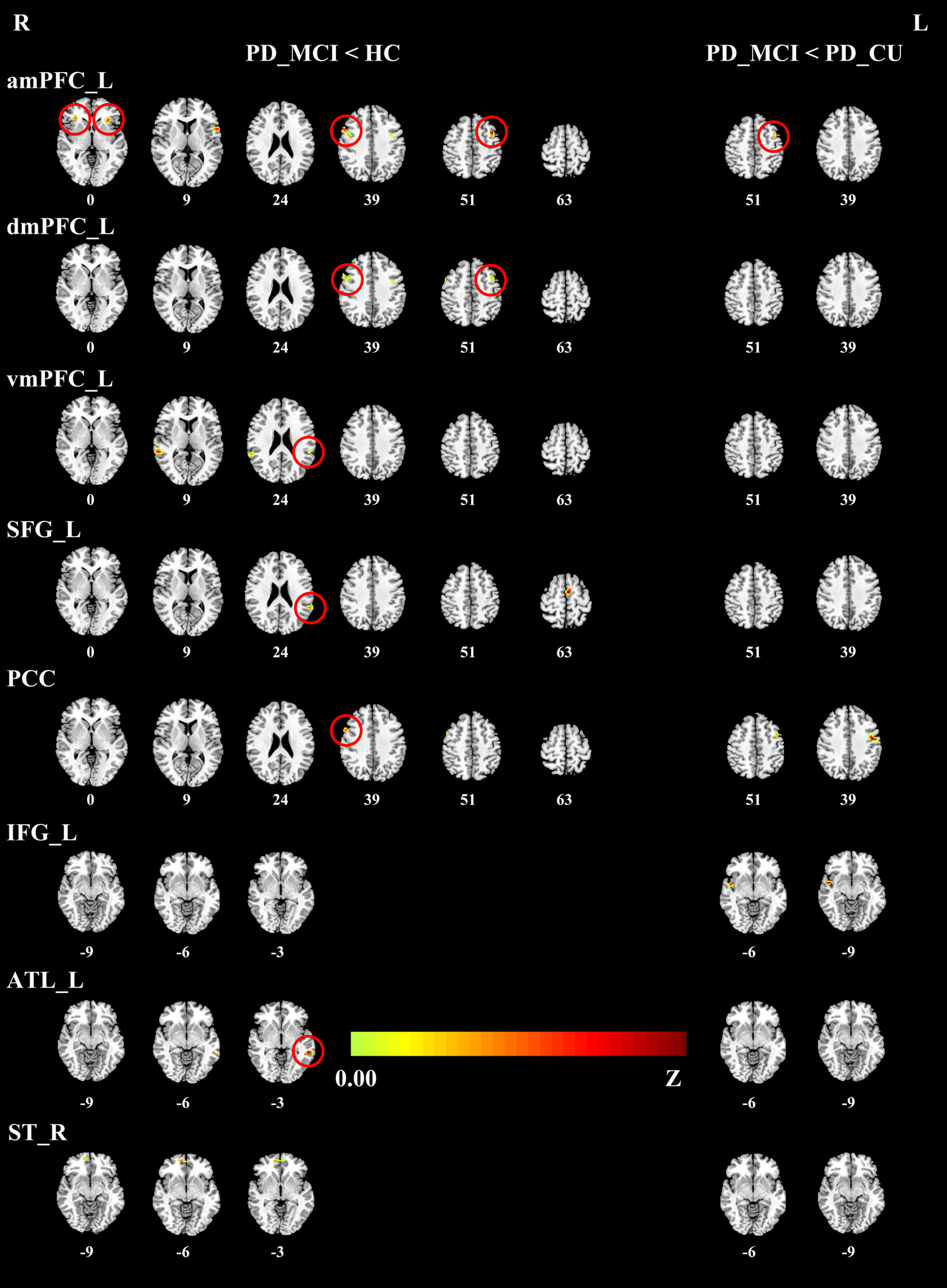

Supplement: Supplementary file 1 [file Image_1.tif]
