# Supplementary material for: Dysfunction of the Default Mode Network in Drug-Naïve Parkinson’s Disease with Mild Cognitive Impairments: A Resting-State fMRI Study
Source: Front Aging Neurosci. 2016 Oct 26;8:247. doi: 10.3389/fnagi.2016.00247 (PMC5080293; doi:10.3389/fnagi.2016.00247)
Supplement: Supplementary file 2 [file Table_1.DOCX]

Supp. Table 1 Anatomical regions used as DMN nodes for seed-based connectivity analyses

| Region | MNI coordinate  (x, y, z)  Left | | | MNI coordinate  (x, y, z)  Right | | |
| --- | --- | --- | --- | --- | --- | --- |
| Anterior medial prefrontal cortex | -8 | 56 | 14 | - | - | - |
| Dorsal medial prefrontal cortex | -8 | 50 | 34 | - | - | - |
| Ventral medial prefrontal cortex | -2 | 44 | -12 | - | - | - |
| Superior frontal gyrus | -8 | 20 | 62 | - | - | - |
| Inferior frontal gyrus | -42 | 26 | -14 | 50 | 32 | -6 |
| Posterior inferior parietal lobule | -50 | -60 | 28 | 58 | -60 | 28 |
| Precuneus | -2 | -60 | 50 | - | - | - |
| Posterior cingulate cortex | -2 | -48 | 28 | - | - | - |
| Temporal parietal junction | -44 | -52 | 22 | 44 | -58 | 18 |
| Anterior temporal lobe | -52 | -10 | -20 | 52 | -4 | -16 |
| Superior temporal sulcus | -60 | -28 | -4 | 50 | -36 | 4 |
| Hippocampal formation | -26 | -8 | -24 | 24 | -14 | -22 |

Keys: MNI, Montreal Neurological Institute.
