# Supplementary material for: Dysfunction of the Default Mode Network in Drug-Naïve Parkinson’s Disease with Mild Cognitive Impairments: A Resting-State fMRI Study
Source: Front Aging Neurosci. 2016 Oct 26;8:247. doi: 10.3389/fnagi.2016.00247 (PMC5080293; doi:10.3389/fnagi.2016.00247)
Supplement: Supplementary file 3 [file Table_2.DOCX]

Supp. Table 2 Differences in functional connectivity among PD subgroups and normal subjects with global signal regression

| Seed region | P value | | Size | Connected location | T | MNI coordinate  (x, y, z) | | |
| --- | --- | --- | --- | --- | --- | --- | --- | --- |
| **HC group > PD-MCI group** | | | | | | | | |
| Left anterior medial prefrontal | | | | | | | | |
| **Cluster 1** | | **0.029** | **74** | **Left middle frontal** | **4.82** | **-39** | **9** | **54** |
| **Cluster 2** | | **0.053** | **63** | **Right precentral** | **4.50** | **57** | **12** | **42** |
| **Cluster 3** | | **0.104** | **51** | **Right insula** | **4.21** | **33** | **30** | **-3** |
| Cluster 4 | | 0.199 | 40 | Left inferior frontal | 4.45 | -60 | 9 | 9 |
| **Cluster 5** | | **0.223** | **38** | **Left insula** | **4.45** | **-30** | **27** | **3** |
| Left dorsal medial prefrontal | | | | | | | | |
| **Cluster 1** | | **0.060** | **61** | **Right precentral** | **4.60** | **54** | **9** | **42** |
| **Cluster 2** | | **0.075** | **57** | **Left middle frontal** | **4.30** | **-39** | **9** | **54** |
| Left ventral medial prefrontal | | | | | | | | |
| Cluster 1 | | 0.005 | 110 | Right superior temporal | 4.90 | 60 | -39 | 9 |
| **Cluster 2** | | **0.032** | **72** | **Left inferior parietal** | **4.67** | **-66** | **-39** | **18** |
| Cluster 3 | | 0.073 | 57 | Left middle frontal | 5.39 | -24 | 39 | -21 |
| Left superior frontal | | | | | | | | |
| Cluster 1 | | 0.022 | 72 | Left supplementary motor area | 4.76 | -6 | -3 | 60 |
| **Cluster 2** | | **0.048** | **59** | **Left superior temporal** | **4.11** | **-54** | **-45** | **21** |
| Posterior cingulate cortex | | | | | | | | |
| **Cluster 1** | | **0.039** | **68** | **Right precentral** | **5.23** | **54** | **9** | **36** |
|  | |  |  | **Right middle frontal** | **4.60** | **42** | **3** | **60** |
| Left anterior temporal | | | | | | | | |
| **Cluster 1** | | **0.264** | **35** | **Left middle temporal** | **3.86** | **-57** | **-39** | **-3** |
| Right superior temporal | | | | | | | | |
| Cluster 1 | | 0.013 | 95 | Right medium frontal | 4.42 | 12 | 54 | -6 |
| **PD-CU group > PD-MCI group** | | | | | | | | |
| Left anterior medial prefrontal | | | | | | | | |
| **Cluster 1** | | **0.318** | **32** | **Left middle frontal** | **4.17** | **-39** | **9** | **57** |
| Left inferior frontal | | | | | | | | |
| Cluster 1 | | 0.037 | 68 | Right superior temporal | 4.26 | 48 | 0 | -9 |
| Posterior cingulate cortex | | | | | | | | |
| Cluster 1 | | 0.002 | 125 | Left precentral | 4.32 | -45 | -6 | 39 |

A voxel-based comparison of z-value maps among the PD patients with mild cognitive impairment (PD-MCI), PD patients with unimpaired cognition (PD-CU), and HC was performed by using a design model of one-way ANOVA with age, sex and education as covariates, followed by post hoc two-sample t-tests. The significance threshold was set at p < 0.001. Family-wise error (FWE) correction for multiple comparisons was also conducted at the cluster level. The parts of results in **bold** are the same with the results (in Table 2) with 8 nuisance covariates without the global signal regression.

Keys: MNI, Montreal Neurological Institute.
