# Supplementary material for: Dysfunction of the Default Mode Network in Drug-Naïve Parkinson’s Disease with Mild Cognitive Impairments: A Resting-State fMRI Study
Source: Front Aging Neurosci. 2016 Oct 26;8:247. doi: 10.3389/fnagi.2016.00247 (PMC5080293; doi:10.3389/fnagi.2016.00247)
Supplement: Supplementary file 4 [file Table_3.DOCX]

Supp. Table 3 Neuropsychological performance results for healthy controls and Parkinson’s disease patients

| Z score | Controls | | PD-CU | | PD-MCI | | P value^1^ | P value^2^ |
| --- | --- | --- | --- | --- | --- | --- | --- | --- |
|  | ‾X | SD | ‾X | SD | ‾X | SD |  |  |
| DOT-A | 0.000 | 0.397 | 0.251 | 0.480 | -0.322 | 0.492 | 0.003* | 0.002^#^ |
| Golden Stroop Test | 0.000 | 0.605 | 0.287 | 0.689 | -0.369 | 0.439 | 0.013* | 0.004^#^ |
| VFT | 0.000 | 0.354 | 0.216 | 0.400 | -0.278 | 0.388 | 0.003* | 0.001^#^ |
| CDT | 0.000 | 0.496 | 0.262 | 0.526 | -0.337 | 0.322 | 0.003* | 0.001^#^ |
| HVLT-R total | 0.000 | 0.374 | 0.212 | 0.756 | -0.274 | 0.520 | 0.062 | 0.049^#^ |
| BVMT-R | 0.000 | 0.361 | 0.326 | 0.716 | -0.396 | 0.608 | 0.003* | 0.006^#^ |
| WAIS-RC | 0.000 | 0.666 | 0.383 | 0.667 | -0.493 | 0.531 | 0.001* | <0.000^#^ |
| BNT | 0.000 | 0.479 | 0.316 | 0.546 | -0.406 | 0.358 | <0.000* | <0.000^#^ |
| BLO | 0.000 | 0.540 | 0.141 | 0.477 | -0.182 | 0.338 | 0.171 | 0.040^#^ |
| CCT | 0.000 | 0.214 | 0.287 | 0.514 | -0.370 | 0.314 | <0.000* | <0.000^#^ |

^#^ and * indicate significant difference

^1^ Comparison among PD-MCI, PD-CU patients, and control subjects

^2^ Comparison between PD-CU and PD-MCI patients

Keys: DOT-A, adaptive digit ordering test; VFT, verbal fluency test; CDT, clock drawing test; HVLT-R, the Hopkins verbal learning test-revised; BVMT-R, the brief visuospatial memory test revised; WAIS-RC, Wechsler intelligence scale for adult-Chinese revised; BNT, Boston naming test; BLO, Benton Line Orientation; CCT, clock copying test.
