# Supplementary material for: Dysfunction of the Default Mode Network in Drug-Naïve Parkinson’s Disease with Mild Cognitive Impairments: A Resting-State fMRI Study
Source: Front Aging Neurosci. 2016 Oct 26;8:247. doi: 10.3389/fnagi.2016.00247 (PMC5080293; doi:10.3389/fnagi.2016.00247)
Supplement: Supplementary file 5 [file Table_4.DOCX]

| Regions | HC | PD-CU | PD-MCI | P^1^ | P^2^ | P^3^ |
| --- | --- | --- | --- | --- | --- | --- |
| HF_L-SFG_L | 0.233 ± 0.177 | 0.146 ± 0.229 | 0.083 ± 0.240 | 0.114 | 0.043 | 0.410 |
| HF_L-IFG_L | 0.380 ± 0.217 | 0.329 ± 0.252 | 0.191 ± 0.246 | 0.072 | 0.024^#^ | 0.109 |
| HF_L-IFG_R | 0.311 ± 0.202 | 0.265 ± 0.245 | 0.225 ± 0.256 | 0.549 | 0.282 | 0.627 |
| PCC-pIPL_R | 0.827 ± 0.254 | 0.776 ± 0.246 | 0.608 ± 0.301 | 0.057 | 0.019^#^ | 0.081 |
| ATL_L-IFG_L | 0.547 ± 0.200 | 0.508 ± 0.256 | 0.329 ± 0.244 | 0.024 | 0.008^#^ | 0.034^#^ |
| ATL_L-IFG_R | 0.413 ± 0.200 | 0.349 ± 0.294 | 0.236 ± 0.276 | 0.138 | 0.048 | 0.217 |
| ATL_R-IFG_L | 0.420 ± 0.243 | 0.416 ± 0.351 | 0.181 ± 0.384 | 0.067 | 0.034^#^ | 0.044^#^ |
| ATL_R-IFG_R | 0.474 ± 0.199 | 0.420 ± 0.260 | 0.260 ± 0.274 | 0.039 | 0.012^#^ | 0.068 |

Supp. Table 4 Means and SDs of z values for all participants.

^#^ indicates significant difference

^1^ Comparison among PD-MCI, PD-CU patients, and control subjects

^2^ Comparison between HC and PD-MCI patients

^3^ Comparison between PD-CU and PD-MCI patients

Keys: HC, healthy control; PD, Parkinson’s disease; PD-CU, cognitive unimpaired PD patients; PD-MCI, mild cognitive impaired PD patients; HF, hippocampal formation; SFG/IFG, superior/inferior frontal gyrus; PCC, posterior cingulate cortex; pIPL, posterior inferior parietal lobule; ATL, anterior temporal lobe; L, left; R, right.
